# 

**Published:** 1892-12-17

**Authors:** 


**Notices of Births, Deaths, and Marriages,** are inserted in this column at a charge of 2s. 6d. for an announcement not exceeding four lines.

### NOTICE TO CORRESPONDENTS.

All MS., Letters, Books for Review, and other matters intended for the Editor should be addressed The Editor, The Lodge, Porchester Square, London, W.

The Editor cannot undertake to return rejected M.S., even when accompanied by stamped directed envelope.

**Notice to Advertisers and Subscribers.**

All Advertisements, Orders for Copies of The Hospital, and Business Communications should be addressed to The Publisher, *not to the Editor*, The Hospital, 140, Strand, W.C.

The Cost of Subscription, post free, is as follows:—Per week, 2½d.; per quarter, 2s. 6d.; per half-year, 5s.; per annum, 8s. 6d. Foreign:—Per annum, 10s. 8d.; payable, in all cases, in advance.

**"Burdett's Hospital Annual," 1893.**

Fourth year of publication. 700 pp. Boards, gilt lettering. A most complete guide to British, Colonial, Indian, and American Hospitals, Dispensaries, Nursing and Convalescent Institutions, and Asylums. Price 5s. Published by The Scientific Press (Limited), 140, Strand, W.C.

**NOTICE.—The Index for Vol. XII. (ending September, 1892) is on sale at the Office of this Journal, price 2½d., post free.**

### Books Received.

G. Putnam

"Hygenic Measures in Relation to Infectious Diseases." By G. H. Nuttall, M.D.

MacMillan and Co.

"Finger Prints." By Francis Galton, F.R.S.

Bailliere, Tindall, and Cox.

"Symptoms and Physical Signs. A Formulary for Clinical Note-taking." By William Ewart, M.D.

"Aids to Gynæcology." By A. Gubb, M.D.

"The Hygiene of the Ear." By Vincenzo Cozzolino. Translated by J. Erskin, M.B.

### The Student of Medicine.

[All communications for this Supplement should be addressed to The Editor of The Hospital, at 140, Strand, with the words, "Students' Column," written in the left hand top corner of the envelope.]

### APPOINTMENTS.

Anderson, F. T., L.R.C.P., L.R.C.S.Edin., appointed Medical Officer for the No. 4 District of the Alton Union. Andrew, F. W., L.R.C.P.Lond., M.R.C.S., appointed Medical Officer to the Hendon Local Board. Bullmore, William King, M.D.St. And., M.R.C.S.Eng., appointed Medical Officer of Health for the Falmouth Urban Sanitary District. Callaghan, Thomas, L.R.C.P., L.R.C.S.Edin., L.F.P.S.Glasg., appointed Medical Officer for the No. 6 District of the Cork Dispensary. Carruthers, J. Ferguson, M.B., C.M.Edin., appointed Medical Officer and Public Vaccinator for the Rivesly Rural District of the Horncastle Union. Crook, Arthur, L.R.C.P.Lond., M.R.C.S.Eng., re-appointed Medical Officer for the Boys' Home of the Norwich Union. Dolamore, W. H., L.R.C.P., M.R.C.S., L.D.S., appointed Second Dental Surgeon to the Westminster Hospital. Douglas, Dr. N. G., appointed Medical Officer for the Western District of the Scarborough Union. Eden, William A., L.S.A., appointed Assistant Medical Officer of the Workhouse and Infirmary of the St. George's Union. Elliott, Harry Scott, M.R.C.S.Eng., L.R.C.P., L.S.A.Lond., appointed Assistant House Physician to St. George's Hospital, London. Humphreys, John, L.R.C.P. Edin., L.F.P.S.Glasg., appointed Medical Officer for the Trawsfynydd District of the Festiniog Union. Latimer, H. A., M.R.C.S., appointed Senior Surgeon to the Swansea General Hospital. Lory, Dr., appointed Medical Officer for the Grinley-on-the-Hill District of the Retford Union. Lys, Henry Grabham, M.D.Lond., M.R.C.S., appointed Medical Officer for the Out-Patients to the Royal Victoria Hospital, Bournemouth. Malvin, Mark, L.R.C.P.Edin., M.R.C.S.Eng., appointed Medical Officer for the Eastern Division of Scarborough and Medical Officer for the Scarborough Workhouse. Oldacres, C. E., M.R.C.S., L.R.C.P.E., appointed Medical Officer of Health for the Borough of Daventry. Reed, Henry A., L.R.C.P.Lond., M.R.C.S., appointed Medical Officer of Health for Hamilton, Tasmania. Richardson, R. T., M.R.C.S.Eng., L.S.A.Lond., appointed Medical Officer of Health for the Trowbridge Urban Sanitary District. Roberts, Sidney John, L.R.C.P.Lond., M.R.C.S.Eng., appointed one of the Resident Surgeons to the Birmingham General Dispensary. Sinclair, Wm., M.B., C.M.Aberd., appointed Assistant Surgeon to the Royal Infirmary, Aberdeen. Smith, Mr., appointed Medical Officer of Health for the Ampthill Urban Sanitary Authority. Smithies, Joseph Jackson, L.F.P.S.Glasg., L.R.C.P.Edin., re-appointed Medical Officer of Health for the Gisburn District of the Clitheroe Union. Spowart, William Ribton, B.A., M.D. Dub., L.R.C.P.I., re-appointed Medical Officer for the No. 8 District of the Norwich Union. Stacey, John Herbert, L.R.C.P., L.R.C.S.Edin., re-appointed Medical Officer for the No. 6 District of the Norwich Union. Sykes, John Frederick, M.B., B.Sc., re-appointed Medical Officer of Health for the Parish of St. Pancras. Tetley, F. Harrison, L.R.C.P., L.R.C.S.Edin., appointed Medical Officer of Halth for the Wangford Urban Sanitary Authority. Thomas, J. T., L.R.C.P.I., L.R.C.S.Edin., appointed Medical Officer for the Magor Sanitary District of the Newport (Mon.), Union. Townsley, Dr., appointed Medical Officer of Health to the Ardsley Local Board. Turner, J. A., C.M.Edin., D.P.H.Camb., re-appointed Medical Officer of

W. POWELL PRICE, Secretary.

**THE STUDENT OF MEDICINE—(*continued*).**

Health to the Melton Mowbray Local Board. West, J. T., M.B., C.M.Aberd., appointed Non-resident House Physician and Surgeon to the Hospital for Sick Children, Aberdeen. Williams, Mr. G. R., appointed Medical Officer for the Fourth District of the Henstead Union. Woodhouse, William, M.R.C.S.Eng., L.S.A., re-appointed Medical Officer for the No. 1 District of the Norwich Union. Young, Meredith, M.B., C.M.Edin., Medical Superintendent of Brighouse Joint Hospital, appointed Medical Officer of Health for Rastrick.

### VACANCIES.

The following vacancies are announced this week, the amount of salary in each case being given after the name of the institution:

### Resident Medical Officers.

Belgrave Hospital for Children, 79, Gloucester Street, S.W. House Surgeon. Board, lodging, fuel, and light found. Applications, endorsed on envelope "House Surgeon," to the Honorary Secretary, by December 16th.

Brecon Infirmary, 6, Bulwark, Brecon, South Wales. Resident House Surgeon; unmarried; doubly qualified. Salary £70 per annum, with furnished apartments, board, attendance, fire, and gas. Applications to W. Powell Price, Secretary, by December 26th.

Bridgwater Infirmary. House Surgeon. Salary £80 per annum, with board and residence. Applications to Mr. John Coombs, Honorary Secretary, by January 1st, 1893.

Central London Ophthalmic Hospital, Gray's Inn Road, W.C. House Surgeon. Applications to the Secretary, by December 20th.

Chichester Infirmary. House Surgeon; qualified and registered. £80, with board, lodging, and washing. Applications by January 24th, 1893.

Clutton Union. Medical Officer and Public Vaccination Officer for the Chew Magna District. Salary £66 per annum and vaccination fees. Applications to E. H. Perrin, Solicitor and Clerk to the Guardians, Temple Cloud, near Bristol, by December 14th.

General Hospital, Birmingham. Assistant House Surgeon. Board, residence, and washing provided. Applications to the House Governor, by December 31st.

Infirmary for Consumption and Diseases of the Chest and Throat, 26, Margaret Street, Regent Street, W. Three Visiting Physicians. Applications to William Henry Johnson, Secretary.

Manchester Southern and Maternity Hospital. Resident House Surgeon. Applications to the Honorary Secretary, George William Fox, 53, Princess Street, Manchester.

Mile End Old Town. Dispenser of Medicines at the Infirmary and Outdoor Dispensaries, Bancroft Road, E., between 25 and 45 years of age. Salary £130 per annum, and dinner daily. Applications to William Thacker, Clerk, Guardians' Offices, Bancroft Road, Mile End, E., by December 15th.

North Staffordshire Infirmary and Eye Hospital, Hartshill, Stoke-on-Trent. House Surgeon; doubly qualified. Salary £120 per annum, increasing £10 yearly, with furnished apartments, board, and washing. Applications to the Secretary, by January 21st, 1893.

Northumberland County Lunatic Asylum, Morpeth. Assistant Medical Officer; unmarried. Salary £120 per annum, increasing £10 yearly to £150, with furnished apartments and board and lodging. Applications to Dr. McDowall, at the Asylum, by December 15th.

Royal Albert Hospital, Devonport. Assistant House Surgeon. Board, lodging, and washing provided. Applications to the Chairman of the Medical Committee, by December 16th.

Stockport Infirmary. House Surgeon; doubly qualified. Salary £100 per annum, with board and apartments. Applications to Lieutenant-Colonel S. W. Wilkinson, Honorary Secretary, by December 20th.

Victoria Hospital, Folkestone. House Surgeon; unmarried. Salary £80 for the first year, with an increase of £10 each year for the two following years, with board and residence. Applications to the Secretary, by December 13th.

Westminster General Dispensary, 9, Gerrard Street, Soho. Resident Medical Officer. Appointment for one year. Applications to J. J. Johnson, Secretary, by December 15th.

### Non-Resident Medical Officers.

Killarney District Lunatic Asylum. Assistant Medical Officer. Salary £100 per annum, with allowances valued at £100 yearly. Candidates must be unmarried, and not over 30. Applications to Dr. L. T. Griffin, Resident Medical Superintendent. Election on January 19th.

Parish of Tongue, Sutherlandshire. Medical Officer. Salary £165 per annum; house available. Applications to John Murray, Inspector of the Poor, Tongue, Sutherlandshire.

### THE STUDENT OF MEDICINE—(*continued*).

Royal Westminster Ophthalmic Hospital, King William Street, West Strand. Clinical Assistants. Applications to the Secretary, by December 31st.

St. Andrews University, Dundee. Demonstrator of Anatomy. Salary £120 per annum. Applications to the Secretary, by December 17th.

University of Edinburgh. Examiner in each of the following subjects: (1) Anatomy, (2) Chemistry and Laboratory Work for the First B Sc. Examination in Public Health, (3) Midwifery, (4) Practice of Physic, and (5) Botany. The salary in each of the departments Nos. 1, 3, 4, and 5 is £75 a year, and in No. 2 £90 a year, with an allowance of £10 per annum for travelling and other expenses in the case of Examiners not resident in Edinburgh or the immediate neighbourhood. Applications to M. C. Taylor, Secretary, by January 9th, 1893.

### PASS LISTS.

**Royal College of Physicians and Surgeons, Edinburgh, and Faculty of Phyicians and Surgeons, Glasgow.**

The quarterly examinations for the triple qualification in Edinburgh took place in October and November with the following results:—

First Examination.—Of 53 candidates, the following 20 passed: A. Munro, Edinburgh; W. H. O. Garde, County Cork; C. E. Conran, Madras; C. N. Winch, Chatham; R. K. Imeary, Durham; J. C. Glen, Middlesboro'; D. M. Atkinson, Yorkshire; G. R. Twomey, Cork; A. Whitehead, Burnley; R. R. Dobson, Glasgow; T. O'Callaghan, County Cork; J. W. Spawforth, Winthorp; H. H. Warren, Armagh; A. H. Bell, Jamaica; C. B. Johnstone, Manchester; W. P. McGrenahan, Dublin; G. H. Pearce, Whitby; W. A. Mushet, Paris; A. R. Wilkinson, Lancashire; and E. MacCormack, Roscommon. Of nine candidates who entered for the respective divisions, six passed.

Second Examination.—Of 72 candidates, the following 46 passed: J. O'Leary Driscoll, Bandon; W. T. Wood, County Cork; T. A. Wiltshire Walker, Hyderabad; G. F. Taylor, India; W. Corkey, County Armagh; H. C. Swailes, Yorkshire; W. J. Darbyshire Preston, Rock Ferry; W. H. Ormonde Garde, County Cork; D. F. Blanchard, Armagh; W. Mortimer Sheen, Gravesend; C. H. Roberts, Adelaide; L. J. Quigley, Kings County; R. Geren Barnes, County Tyrone; J. J. Hayes, County Limerick; G. E. Watson, County Durham; W. Pearson, Manchester; A. Whitehead, Burnley; D. Hart Scott, Dumfries; E. Cottew Moore, Portsmouth; H. Bond, Preston; J. Prichard, Carnarvon; E. MacCormack, Roscommon; A. Clark, Sheffield; W. P. McGrenahan, Tipperary; J. Elliot, Edinburgh; Beatrice Garvie, Perth; E. McGarity, Bishop Auckland; W. Heatlie Ferrier, Edinburgh; J. J. FitzGerald, County Cork; Claudiano Jose da Cunha, India; C. Seymour Langley, County Cork; W. Fleming Stevenson, Strabane; E. Fergus Jamison, County Down; Janet McIntyre Kay, Castle Douglas; Martha F. Armitage, Staffordshire; M. Stewart, Ayrshire; D. Y. Clark, Perthshire; W. H. Penrose, Cork; W. A. Deason, St. Helena; C. W. Lawson, Edinburgh; W. P. O'Connor, Cork; J. Cameron, Fife; T. French, County Cork; W. Curtis, Hampshire; C. Nightingale Winch, Chatham; and J. E. Foley, County Clare. Of 41 candidates for the respective divisions, 23 passed.

Final Examination.—Of 106 candidates, the following 59 passed, and were admitted L.R.C.P. and S.E., and L.F.P. and S.G.: I. H. Ross, Demerara; N. G. Douglas, Crewe; J. A. R. Stollmeyer, Trinidad; J. W. Stoker, Durham; G. H. K. Ross, Jamaica; J. T. Helm, Cape Colony; Oswald Horrocks, Manchester; S. B. Haynes, County Cork; J. A. Leishman, India; C. E. Whitcher, London; C. E. Oakeley, Monmouth; R. W. Fell, Durham; F. B. Lewis, Kent; Rosa E. Bale, Devon; Edith G. Collett, Madras; Lilian Trewby, London; J. Wilkie, Newburgh-on-Tay; C. G. Webster, Chandernagore; H. Robins, Jamaica; H. A. E. Noble, Durham; W. S. Harvison, New South Wales; C. A. Kalenberg, Ceylon; E. R. Pollard, Burnley; Mary C. Murdoch, Elgin; A. Clark, Dorset; E. E. Cooper, Great Yarmouth; G. W. Wickham, Winchester; J. O. Bartholomeusz, Ceylon; Edith L. Nicholas, Middlesex; J. Cook, Castlemaine; R. Fair, County Galway; L. C. Murphy, Melbourne; Hirji Jehangir Dadysett, Bombay; Freany Khursedjee Cama, Bombay; F. Brown, Govan; P. F. Evans, Cork; J. Chadwick, Burnley; A. A. Fermie, Calcutta; T. G. G. Symons, Falmouth; F. P. Hill, India; L. Burges, Banffshire; W. Beatt, Cavan; T. W. Waddell, Demerara; D. de Jersey White, Canada; H. L. Murray, Victoria; R. T. Clark, Cumberland; J. H. Wilson, Plymouth; H. Tatley, Liverpool; J. B. Wilson, County Cork; J. J. C. Elmes, Limerick; R. A. Mate, Salop; J. F. O'Meara, Limerick; J. T. McArthur, County Tyrone; A. J. McDonald, South Australia; A. G. Merson, Banffshire; T. M. Martin, Lanarkshire; W. I. Senkler, Canada; M. Jones, North Wales; and R. H. Manson, Darlington. Of 66 candidates who entered for the respective divisions, 31 passed.

